# The Fitting and Furnishing of Wards and Operating Theatres

**Published:** 1906-11-17

**Authors:** 


### THE FITTING AND FURNISHING OF WARDS AND OPERATING THEATRES.

### DISPOSAL OF WASTE.

For the disposal of solid refuse of all kinds, the refuse destructor is the best arrangement. Messrs. Manlove, Alliott and Co., of Nottingham, and Messrs. Meldrum, of Timperley, Manchester, have given a great deal of attention to this subject, and produce very convenient and thoroughly efficient apparatus for the purpose. One great requisite, wherever heat is employed to completely destroy refuse, is that the fumes shall themselves be subject to a very high temperature before the final products are allowed to escape into the atmosphere. Though germs are killed at the temperature of boiling water, provided that that temperature reaches them, the products of combustion, of all refuse containing compounds, which if not broken up by a high temperature, form a bed for any germs that may be in the atmosphere to which they escape from the chimney. This was the reason of the non-success of the early refuse-destructor employed for town's refuse. The matter is arranged by either making a separate combustion chamber, through which the products of combustion pass after leaving the destructor proper, or by forming a very hot flue for the same purpose. The products of combustion should be subjected to a temperature of 1,500° F. at least.

For liquid refuse all drains should be properly trapped outside the building, where the drainage is to the sewers of the town, and there should be facilities for inspecting the flow from each ward to the drains, by means of properly sealed inspection chambers with manholes. For hospitals and institutions in country districts there are now several forms of apparatus made dealing with the sewage on the septic system. A common form is—the whole of the sewage is allowed to pass into a closed chamber filled with crushed "saggers," the refuse from the pottery kilns, from which light and air are excluded, and from there to filter beds, where it is exposed to the air, the effluent, it is claimed, being perfectly innocuous.

### Mortuaries, Post-mortem Rooms, Etc.

Post-mortem tables in modern hospitals are made of glazed fire-clay, or in some cases of enamelled iron, the former being the favourite, and they are provided with drainage channels, leading to a central drain pipe, the end of which may be in a bucket, or may lead to the sewer. The favourite form of post-mortem table is suspended on a substantial pillar, fixed at its centre, and around which it can revolve. Plain slate tables are also employed, on iron supports. The fittings of the lavatories in connection with the post-mortem rooms are of gun metal. The slabs for the bodies waiting and the lavatory basins and sinks are all of glazed fire-clay, the remarks made in connection with the lavatories of operating theatres applying here as well. In the mortuary the dead are laid, in the leading hospitals, on enamelled iron trays, supported on iron frames, and they are easily and quickly removed thence to the mortuary chapel which usually adjoins, and are there placed, in their trays, upon a bier provided for the purpose, which is often decorated with flowers, etc. In several of the London hospitals, the dead are preserved by cold storage methods. The bodies are not frozen. In cold storage language they are "chilled," that is, they are cooled to a temperature at which decomposition is arrested, and they then present the peaceful aspect immediately after death has taken place, so common to even those who have died in pain. Where this arrangement holds there is a cold chamber, forming part of the mortuary, in which the enamelled iron trays are laid on rails, in separate cells, the temperature of the cells being maintained at about 28° F. On the arrival of the friends, the tray containing the particular body is taken out of its cell, and conveyed to the mortuary chapel. At the Victoria Hospital at the London Docks the post-mortem room, the mortuary, and the mortuary chapel adjoin each other, all having terrazzo floors, with the walls glass tiled, the whole of the rooms being well ventilated. The ventilation of post-mortem rooms and operating theatres is a very important matter. Operating theatres in the leading hospitals are all ventilated by some modification of the "Plenum" system, warmed air being brought into the theatre, generally at a low level, and the atmosphere being exhausted by the aid of fans near the roof. There is a complaint that, in some operating theatres the atmosphere becomes very warm after a certain time; but it appears to the writer that this difficulty could be easily arranged, especially where there is a cold storage plant on the ground. The temperature should be under the control of those in the theatre. In some cases this is accomplished by means of rods, ending in levers in the theatre, working valves controlling the supply of steam to the heating apparatus. A better plan appears to be that which is employed at some technical colleges. Air is delivered to the room by two ducts, the two forming one duct where the air enters the room. Where the two ducts join is a valve, of simple construction, arranged to admit more air from one duct, while it shuts off the supply to the same extent from the other. One duct supplies warmed air, the other air at the temperature of the atmosphere, and the valve is controlled in the room to be ventilated. For post-mortem rooms Webb's lamps, which are also used for the ventilation of sewers and public conveniences have been employed in some hospitals. The lamps consist of two or more Welsbach mantles, burning illuminating gas in the usual way. The lamp has a hood, which is constructed on the lines of the walls of a cold storage room, the object being to confine the heat given off by the gas burners, and to produce an atmosphere at a temperature of 600° F., through which all the noxious vapours have to pass, and are destroyed on their way to the outer atmosphere. The writer understands that in France they have a system of preserving the dead for a very long time, by injecting a preservative. Mr. Michelli, the Superintendent of the Victoria Hospital at the London Docks informed him that in Paris he was shown a very large number preserved in this manner lying together without any offensive smell whatever.

